# Severe cutaneous drug toxicity following disitamab vedotin treatment for metastatic gastric cancer: a case report

**DOI:** 10.3389/fonc.2024.1504079

**Published:** 2025-01-16

**Authors:** Zhao Luping, Cheng Zhen, Li Piaopiao

**Affiliations:** ^1^ Department of Pharmacy, Dongyang People’s Hospital, Dongyang, Zhejiang, China; ^2^ Department of Medical Oncology, Dongyang People’s Hospital, Dongyang, Zhejiang, China

**Keywords:** disitamab vedotin, case report, severe cutaneous drug toxicity, adverse effects, metastatic gastric cancer

## Abstract

**Background:**

This study reports a case of severe cutaneous toxicity in a patient with metastatic gastric cancer induced by disitamab vedotin, emphasizing the need for careful monitoring and management in such treatments.

**Case presentation:**

A 71-year-old female was admitted to hospital complaining of serious rashes following the first cycle of disitamab vedotin regimen for metastatic gastric cancer. The doctor diagnosedtoxic epidermal necrolysis (TEN) induced by the drug. The patient received high-dose methylprednisolone due to the side effects. This resulted in a gradual improvement of symptoms.

**Conclusion:**

During the use of disitamab vedotin, patients need to be monitored for severe skin toxicity.

## Introduction

Disitamab vedotin is an antibody–drug conjugate(ADC)comprising a monoclonal antibody against human epidermal growth factor receptor 2 (HER2) conjugated via a cleavable linker to the cytotoxic agent monomethyl auristatin E (MMAE) (1).The approval of disitamab vedotin in China is currently limited to treating locally advanced or metastatic urothelial carcinoma with HER2 overexpression that has previously received platinum-containing chemotherapy and locally advanced or metastatic gastric cancer with HER2 overexpression that has undergone at least two prior systemic therapies (2).

Stevens-Johnson syndrome (SJS) and toxic epidermal necrolysis (TEN) is a severe cutaneous adverse reaction characterized by extensive necrosis and exfoliation of the epidermis. The estimated incidence across the disease spectrum is five to six cases per million per year (3, 4). In most cases, SJS/TEN is a severe cutaneous reaction to medications (5). SJS/TEN is predominantly a drug-specific T cell-mediated reaction. The human leukocyte antigen (HLA)-drug-T cell receptor (TCR) engagement results in the activation of drug-specific CD8+ T cells with subsequent release of cytotoxic proteins, resulting in epidermal necrolysis (6–9). The causative medication is typically started between one week to one month (occasionally up to two months) prior to the onset of symptoms (10).

We report a case of severe cutaneous drug toxicity caused by disitamab vedotin, which has not been reported before to our knowledge.

## Case description

We report a 71-year-old woman diagnosed with gastric malignancy with bone metastases on June 16, 2023. The patient received treatment with tislelizumab in combination with trastuzumab for metastatic gastric cancer from July 7, 2023. The dose of tislelizumab was 200mg/d, and the dose of trastuzumab was 400mg/d, both administered every 3 weeks. After completing 14 treatment cycles, which were last used on April 8, 2024.

**Figure 1:**
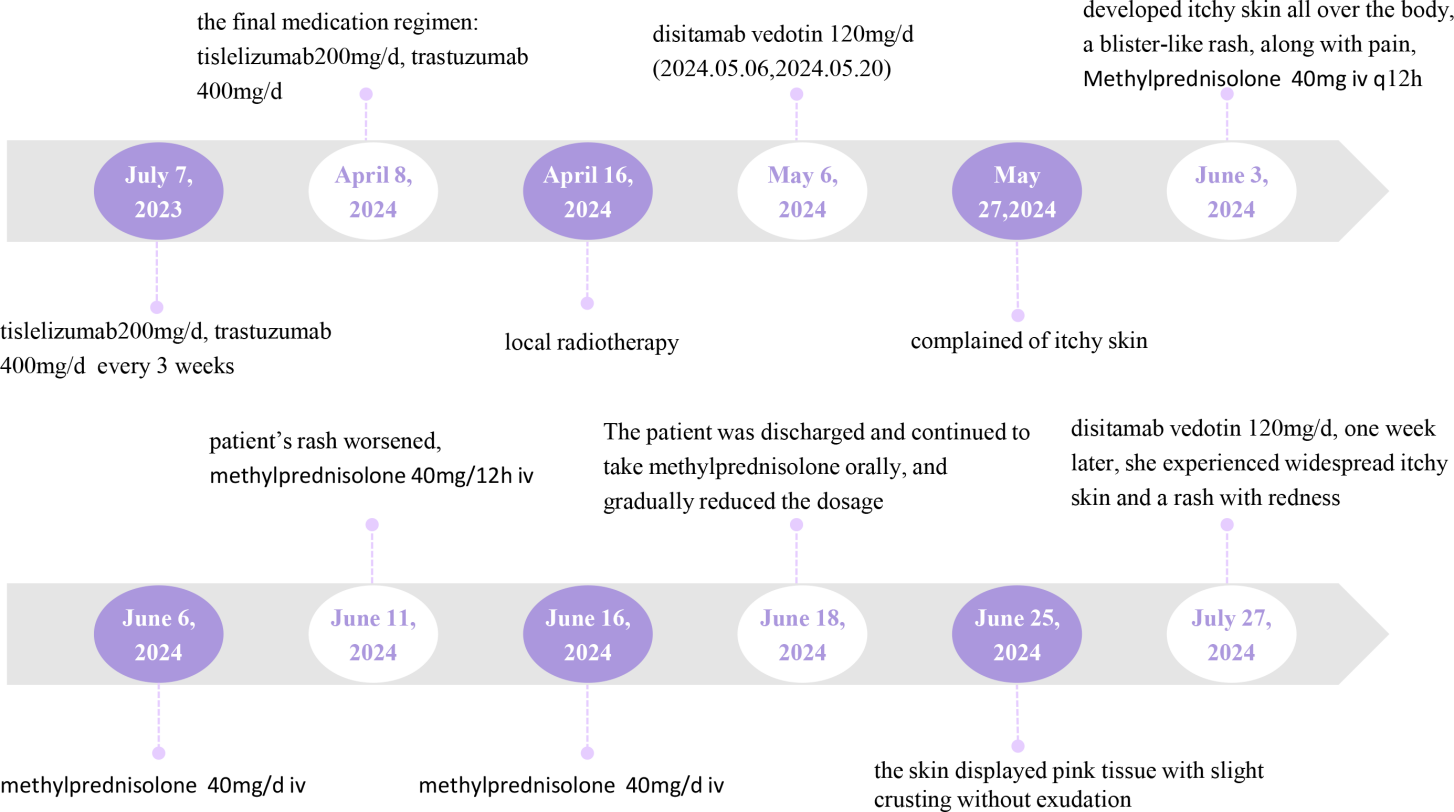
Timeline.

On April 16, 2024, the patient underwent local radiotherapy for a metastatic bone tumor. Following the gastroscopic biopsy, which revealed HER2 (3+), the patient was treated with disitamab vedotin on May 6, 2024. The dosage of disitamab vedotin was 120 mg/d, administered every 2 weeks. **Figure 1** provides a comprehensive illustration of the patient’s detailed treatment process.

**Figure 2:**
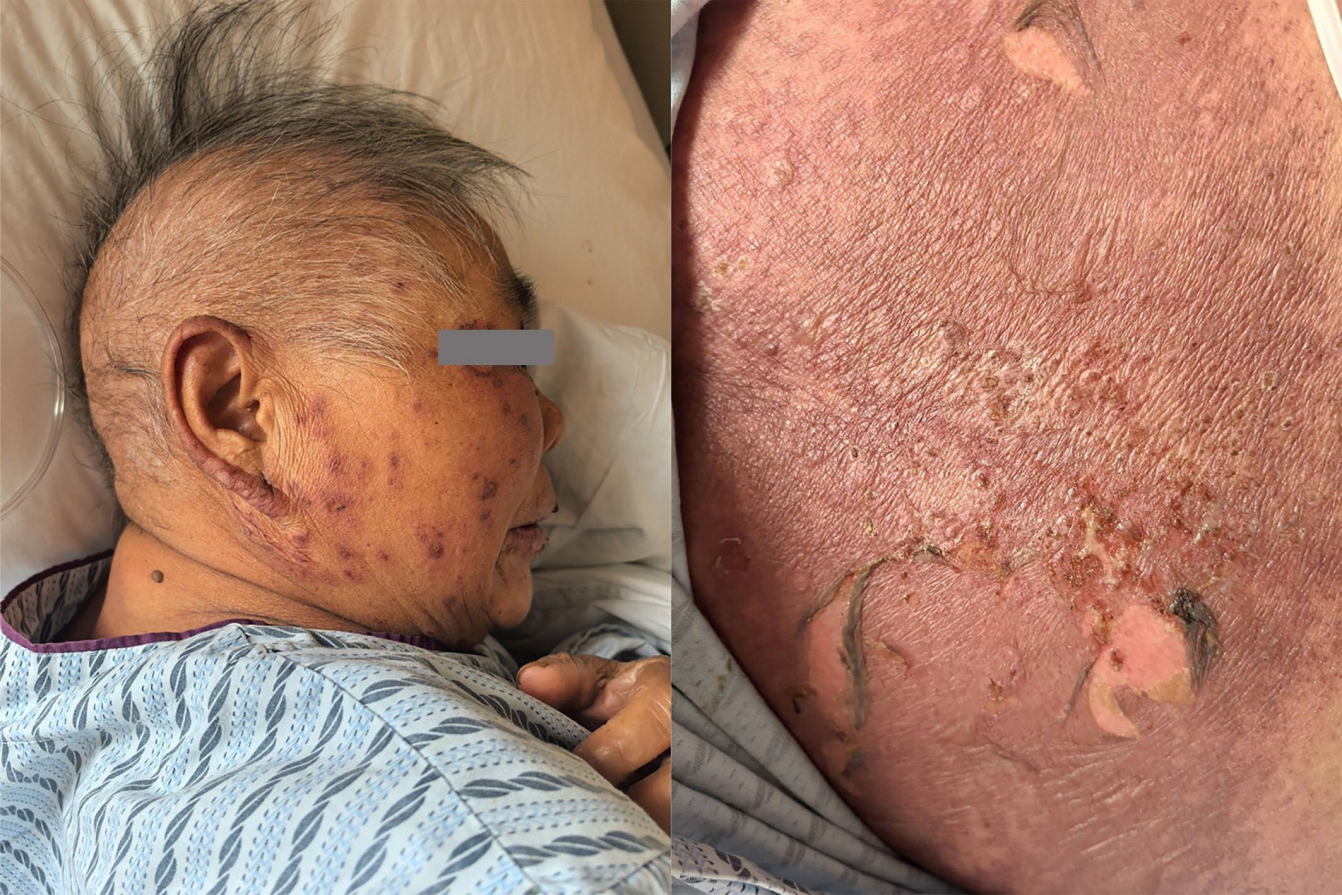
An Initial presentations of generalized erythema, bullae, erosion, and exudation.

On the seventh day after completing two treatment cycles, the patient complained of itchy skin. Then on the eleventh day, the patient developed itchy skin all over the boy, which gradually worsened and was accompanied by a blister-like rash, along with pain (**Figure 2**).

Upon hospital admission, the patient was alert and in good spirits. There was no swelling of the lymph nodes in the bilateral neck and supraclavicular regions. Breath sounds in both lungs were clear, with no dry or wet rales detected. The heart rhythm was regular, and no murmurs were present. The abdomen appeared distended, but no spider nevi or superficial varicose veins were observed. There was no tenderness, rebound tenderness, or palpable masses throughout the abdomen. The liver and spleen were not palpable below the costal margin, and Murphy’s sign was negative. There was no percussion pain in the liver or kidney areas, and both lower limbs were free of edema.

At the time of admission, the patient’s body temperature was normal. Laboratory results indicated a C-reactive protein level of 21.6 mg/L, a neutrophil percentage of 77.5%, and a white blood cell count of 1.64 x 10^9/L, suggesting myelosuppression induced by disitamab vedotin. However, pathology was not obtained because the patient refused a skin biopsy.

**Figure 3:**
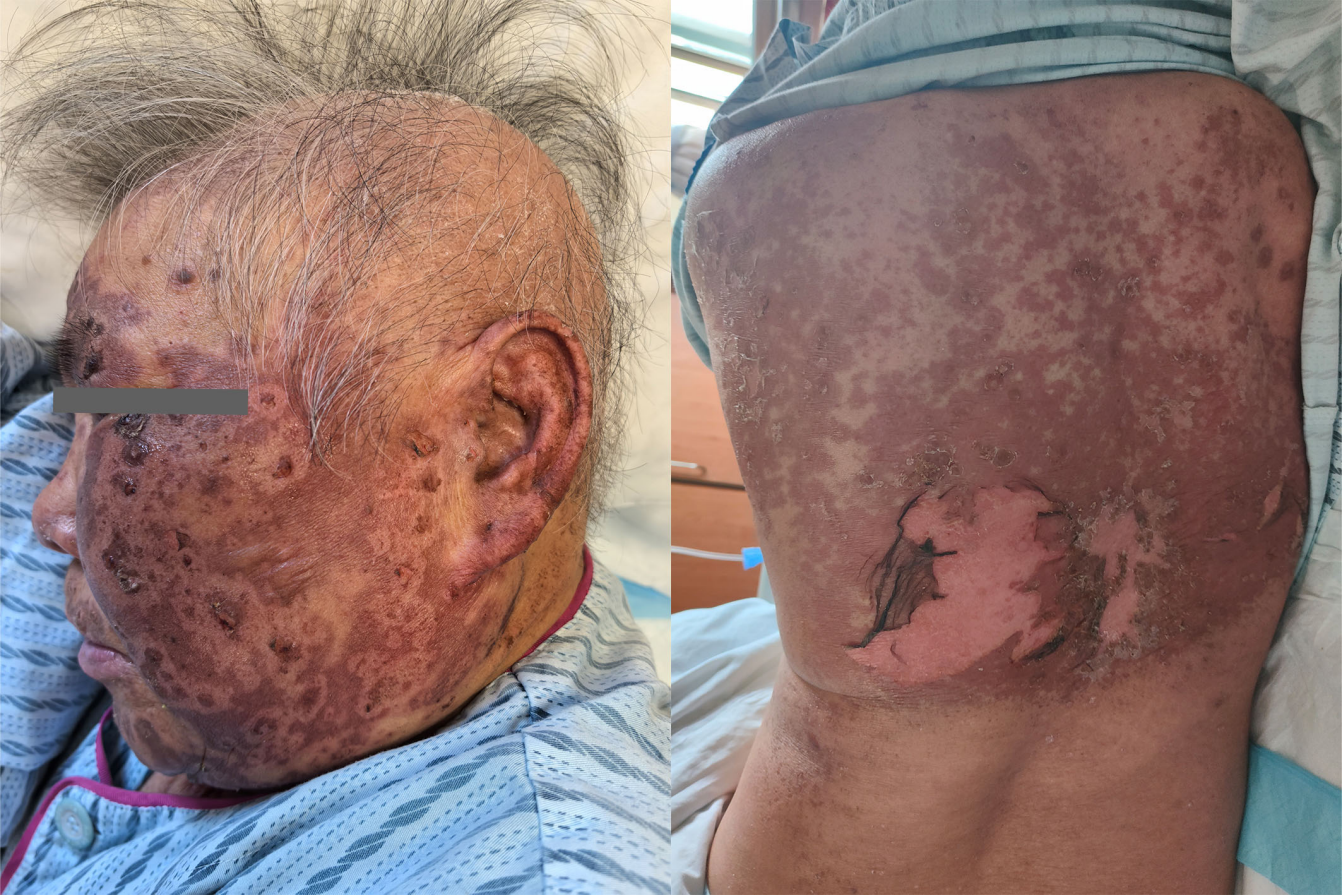
Increased area of erythema.

Based on the patient’s medication history and clinical symptoms, the doctor diagnosed the patient with TEN according to the UK guidelines for the management of Stevens-Johnson syndrome/toxic epidermal necrolysis in adults 2016 (11). The disitamab vedotin was immediately discontinued, and corticosteroid treatment was initiated.Methylprednisolone succinate sodium was administered intravenously at a daily dose of 80 mg. After 3 days of treatment, as the medication dosage was reduced to 40mg daily for 5 days, the patient’s rash suddenly worsened (**Figure 3**). Fortunately, the patient’s eyes and mucous membranes were not affected. The methylprednisolone dose was adjusted to 80mg daily for 5 days.

The patient’s rash improved and the bullae began to dry up, allowing for a reduction of methylprednisolone to 40 mg daily. The patient received a total of 16 days of glucocorticoid treatment during the hospitalization.

**Figure 4:**
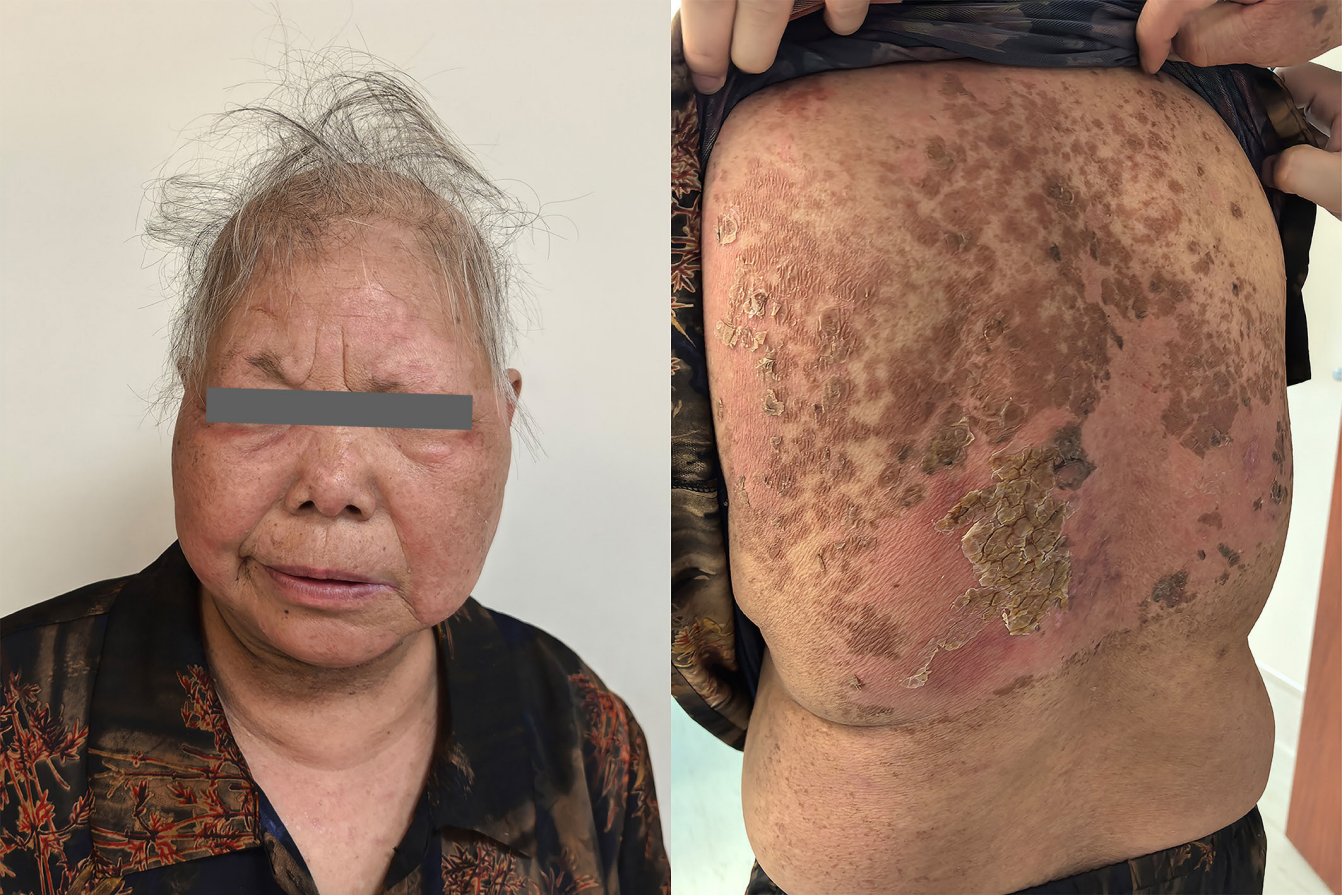
One week after discharge, pink skin tissue on the face and scab on back.

After discharge, the patient continued oral administration of prednisolone tablets at 16 mg daily. One week later, the patient had a follow-up visit, and the skin displayed pink tissue with slight crusting without exudation (**Figure 4**). Seven week later, the patient’s rash had mostly subsided, with only skin pigmented deposition.

On July 2, the patient had a follow-up examination that included upper abdominal and chest CT scans. Compared to the scans taken on April 13, there was a noticeable reduction in the stomach lesions, and the soft tissue mass in the right chest wall had significantly diminished (**Supplementary Figures 1**-**3**).

The patient was administered 120mg of disitamab vedotin on July 27, 2024. However, one week later, she experienced widespread itchy skin and a rash with redness, which improved after receiving symptomatic treatment. Given the patient’s good efficacy and reduced skin adverse reactions after the second administration, we plan to decrease the use of disitamab vedotin.

## Discussion

Tislelizumab, a novel humanized IgG4 monoclonal antibody, exhibits high PD1 affinity. PD-1/PD-L1 immune checkpoint inhibitors (ICIs) have revolutionized cancer therapy but can cause diverse cutaneous immune-related adverse events (IrAEs) (12–17).There have been instances of severe skin adverse reactions reported after 10 cycles of tislelizumab (18).Therefore, we believe that tislelizumab may lead to skin toxicity. However, in our case, one week after disitamab vedotin was re-administered; the patient experienced a recurrence of skin reactions. Disitamab vedotin and tislelizumab demonstrated notable scores of 4 and 2 respectively on Naranjo’s scale. We strongly believe that disitamab vedotin has caused a severe skin adverse reaction.

The mechanism of action of immune checkpoint inhibitors involves restoring the immune system’s ability to target and kill cancer cells by blocking specific proteins at immune checkpoints. This patient has received multiple round of tislelizumab. It is possible that tislelizumab enhances the woman’s sensitivity to treatment with disitamab vedotin (19).

The pathogenesis of severe cutaneous adverse reactions caused by disitamab vedotin remains unclear. Disitamab vedotin is an ADC. ADCs are a new emerging class of highly potent pharmaceutical drugs, which is a great combination of chemotherapy and immunotherapy. They are onsist of three critical elements: (1) an antibody with specificity for an antigen, ideally expressed solely on tumor cells, (2) a cytotoxic agent (or payload) that is designed to cause destruction of the target cell once internalized and released, and (3) a linker molecule that attaches the cytotoxic agent to the antibody (20).Adverse reactions of ADC drugs vary due to their different targets and mechanisms of action. The most commonly reported cutaneous adverse reactions were caused by enfortumab vedotin, which consists of a recombinant humanize Nectin-4 monoconal antibody, an adhesion molecule that plays an important role in cell adhesion by recruiting cadherin and regulating cytoskeletal rearrangement, an enzymatic cleavage linker, and MMAE. While disitamab vedotin is composed of HER-2 monoclonal antibody, cathepsin cleavable linker, and MMAE. Both medications contain the same cytotoxic agent, MMAE. MMAE is an antimitotic agent that exerts its action by blocking the tubulin polymerization process resulting in cell cycle arrest and apoptosis (21). ADCs with MMAE as the payload consistently report Grade 3 or greater hematologic toxicities (≥5% for each toxicity) and peripheral neuropathy (22). We believe that MMAE is not responsible for the skin toxicity observed. The Nectin-4 monoclonal antibody is found in the upper basal layer of normal epidermis, which makes it susceptible to skin toxicity (23). Disitamab vedotin effectively targets HER-2, demonstrating potent anti-tumor effects while simultaneously inhibiting the epidermal growth factor receptor (EGFR) pathway. EGFR and its downstream signaling pathways play a crucial role in various biological processes, including cell proliferation, differentiation, migration, and apoptosis. In skin tissue, EGFR is expressed in keratinocytes, which are found in the basal layer, suprabasal layer, and outer layer of hair follicles. The EGFR pathway is essential for regulating normal growth and differentiation in the epidermis; it stimulates epidermal growth, inhibits differentiation, and accelerates wound healing. However, blocking the EGFR pathway in the skin can trigger inflammatory reactions, leading to various adverse skin conditions (24).

In addition, local radiotherapy may trigger immune-mediated local hypersensitivity (25), and disitamab vedotin may further magnify the hypersensitive response.

SJS and TEN are severe cutaneous adverse reactions that are of major concern because of high mortality rates. The risk of death for patients with identified drug cause was borderline lower than for patients with a reaction of unknown cause (hazard ratio 0.66, 95% CI 0.45-0.96) (26). The cornerstone of the management of these patients during the acute phase is an immediate withdrawal of the responsible drug, supportive care and close monitoring, the prevention and treatment of infections, and a multidisciplinary approach to sequelae (27). Due to the low incidence, there is a lack of systemic experience in managing cutaneous toxicity induced by disitamab vedotin. According to the consensus of experts in the diagnosis and treatment of toxic epidermal necrolysis and the suggestions for the diagnosis and treatment of immune checkpoint inhibitor-related skin adverse reactions, we first selected glucocorticoids for systematic treatment of the patient (28, 29). The patient’s skin was extensively damaged and exudated, but no bacteria was cultured. Studies have shown that the common pathogenic bacteria of TEN are *Staphylococcus aureus*, *Pseudomonas aeruginosa*, and *Enterobacteriaceae organisms* (30). According to the guideline, prophylactic systemic antibiotics should not be given in the absence of clinical signs of a bacterial super infection (progressive redness of the skin, increased or purulent discharge, suspicious vital signs or inflammatory dynamic, or fever) (31). However, the patient had progressive redness of the skin and discharge, and empirical anti-infective therapy with piperacillin/tazobactam 4.5g every 8 hours was administered. After symptomatic treatment, the patient improved. The mechanism of skin toxicity caused by disitamab vedotin is still unclear. We believe a better understanding of the pathogenetic mechanisms will probably lead to a more targeted treatment strategy for these patients in the future.

## Conclusion

In conclusion, this case highlights a unique presentation of severe cutaneous drug toxicity in a patient with metastatic gastric cancer treated with the disitamab vedotin. The management of cutaneous adverse reactions requires close monitoring of patients after administration of the drug, especially in combination with local radiotherapy or immune checkpoint inhibitors.

## Data Availability

The original contributions presented in the study are included in the article/**Supplementary Material**. Further inquiries can be directed to the corresponding author.

## Supplementary material

Supplementary Figure 1 Comparison of hepatogastric lymph nodes on April 13 and July 2.

Supplementary Figure 2 Comparison of gastric CT images on April 13 and July 2.

Supplementary Figure 3 Comparison of right chest CT on April 13 and July 2
