# Supplementary figures and images for: Severe cutaneous drug toxicity following disitamab vedotin treatment for metastatic gastric cancer: a case report
Source: Front Oncol. 2025 Jan 16;14:1504079. doi: 10.3389/fonc.2024.1504079 (PMC11780377; doi:10.3389/fonc.2024.1504079)

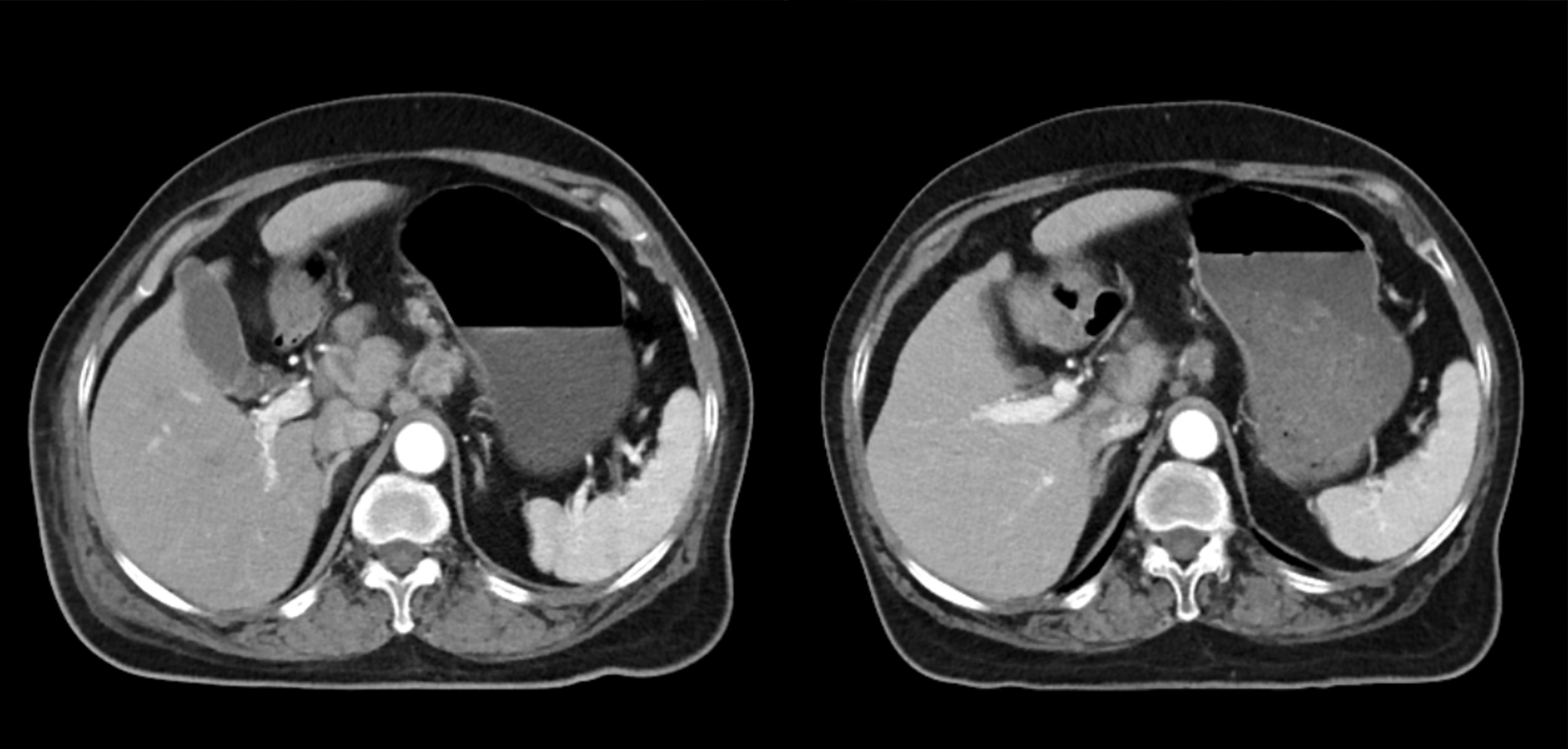

Supplement: Supplementary Figure 1 — Comparison of hepatogastric lymph nodes on April 13 and July 2. [file Image1.jpeg]

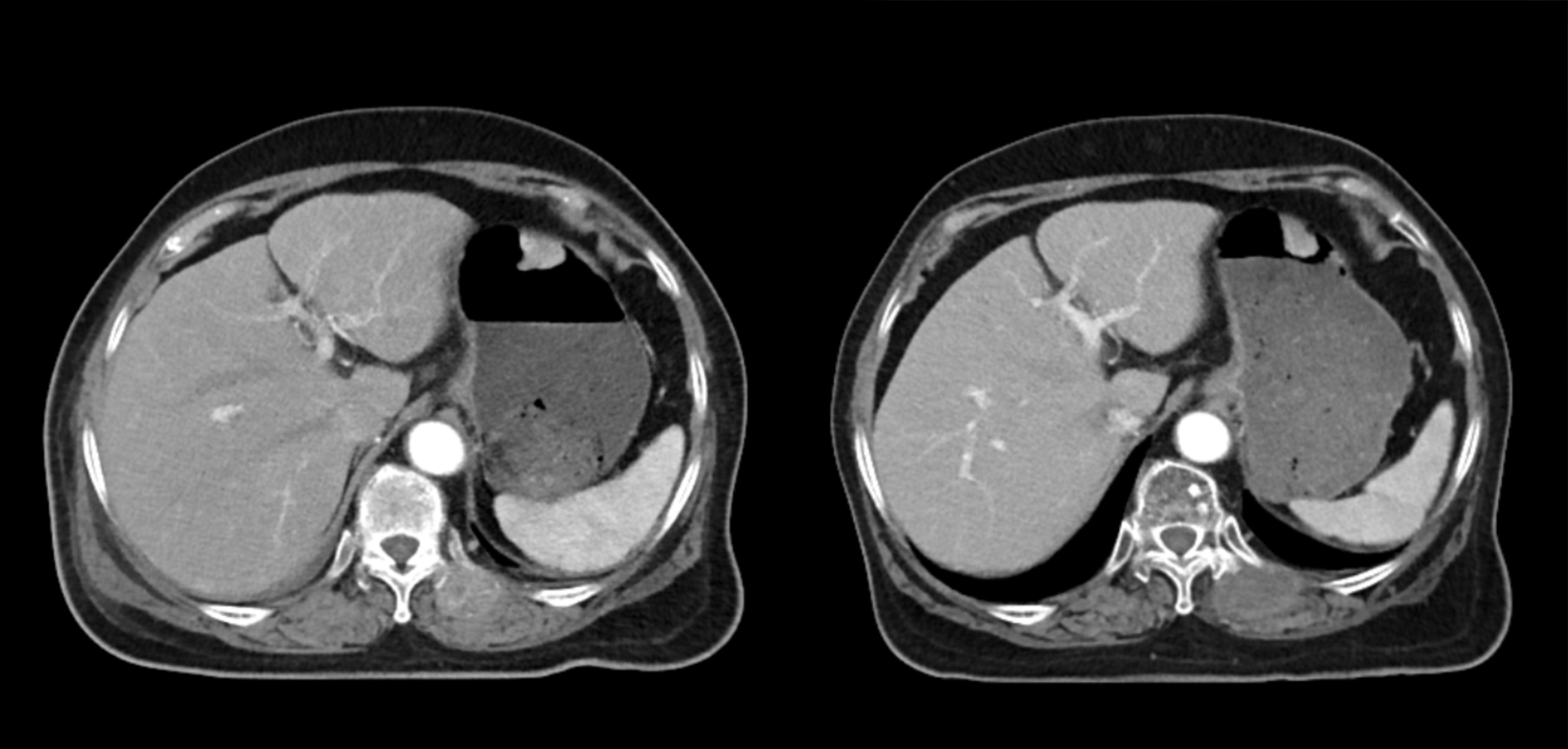

Supplement: Supplementary Figure 2 — Comparison of gastric CT images on April 13 and July 2. [file Image2.jpeg]

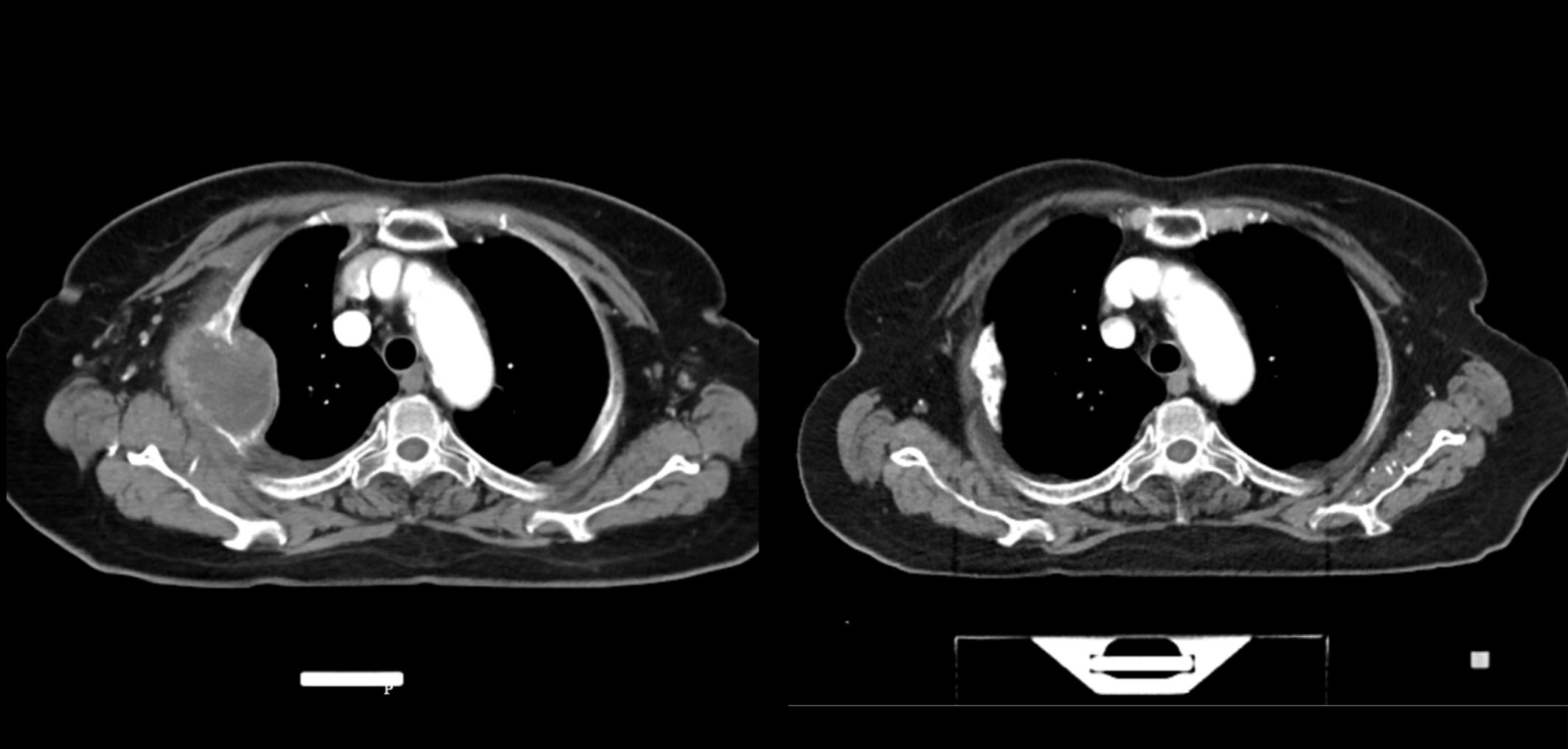

Supplement: Supplementary Figure 3 — Comparison of right chest CT on April 13 and July 2 [file Image3.jpeg]
